# Links between Infections, Lung Cancer, and the Immune System

**DOI:** 10.3390/ijms22179394

**Published:** 2021-08-30

**Authors:** Liviuta Budisan, Oana Zanoaga, Cornelia Braicu, Radu Pirlog, Bogdan Covaliu, Victor Esanu, Schuyler S. Korban, Ioana Berindan-Neagoe

**Affiliations:** 1 Research Center for Functional Genomics, Biomedicine and Translational Medicine, The Iuliu Hatieganu University of Medicine and Pharmacy, 400337 Cluj-Napoca, Romania; liviuta.petrisor@umfcluj.ro (L.B.); braicucornelia@yahoo.com (C.B.); pirlog.radu@umfcluj.ro (R.P.); ioana.neagoe@umfcluj.ro (I.B.-N.); 2 Department of Community Medicine, Public Health and Management, Iuliu Hatieganu University of Medicine and Pharmacy, 400337 Cluj-Napoca, Romania; bogdancovaliu@gmail.com; 3 Faculty of Medicine, Iuliu Hatieganu University of Medicine and Pharmacy, 400012 Cluj-Napoca, Romania; esanuvictor@yahoo.com; 4 Department of Natural Resources and Environmental Sciences, University of Illinois at Urbana-Champaign, Urbana, IL 61801, USA; korban@illinois.edu

**Keywords:** virus, bacteria, lung cancer, immune system

## Abstract

Lung cancer is the leading disease of cancer-related deaths worldwide. Since the beginning of the 20th century, various infectious agents associated with lung cancer have been identified. The mechanisms that include systemic inflammatory pathways as effect of microbial persistence in the lung can secondarily promote the development of lung carcinogenesis. Chronic inflammation associated with lung-cancer infections is known to precede tumor development, and it has a strong effect on the response(s) to therapy. In fact, both viral and bacterial infections can activate inflammatory cells and inflammatory signaling pathways. In this review, an overview of critical findings of recent studies investigating associations between each of viral and bacterial pathogens and lung carcinoma is provided, with particular emphasis on how infectious organisms can interfere with oncogenic processes and all the way through immunity. Moreover, a discussion of the direct crosstalk between lung tumor development and inflammatory processes is also presented.

## 1. Introduction

According to GLOBOCAN 2018, 11.6% of the total cancer cases are diagnosed as lung cancer, the leading cause of cancer death among both men and women worldwide, with 18.4% of the total cancer deaths [1]. Following the dietary factors and smoking, infectious diseases are the third leading causes of cancer worldwide, wherein the percent of cancers associated with pathogenic microorganisms is estimated at 16.1% [2]. For example, it is well-known that pulmonary infections contribute to lung cancer complications [3]. Moreover, post-obstructive pneumonia is negatively associated with lung cancer therapy and the overall survival of cancer patients [4].

**Figure 1:**
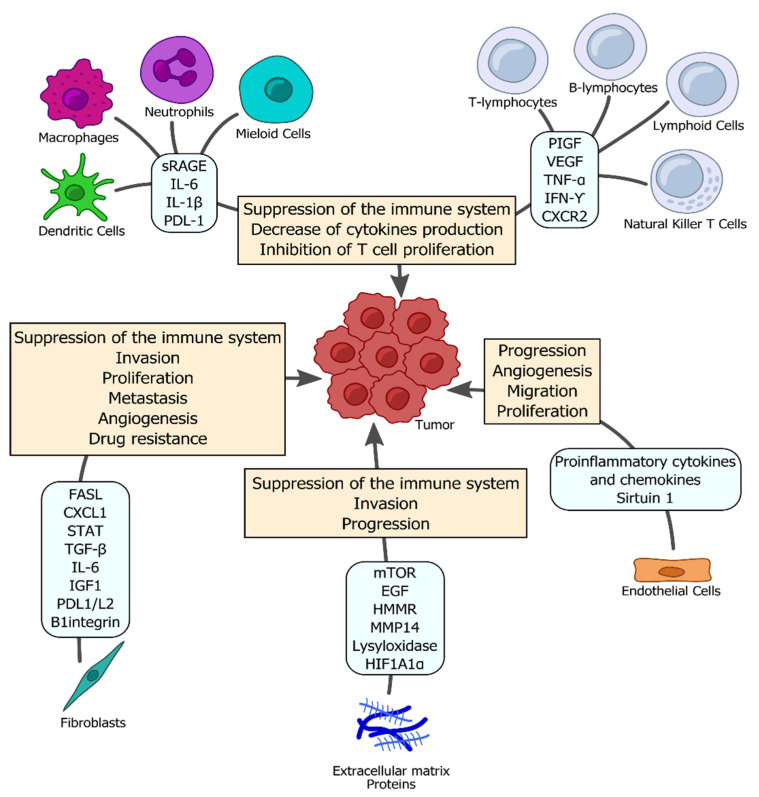
The tumor microenvironment in lung cancer. An effective development of tumors at the primary and metastatic sites depends on the surrounding environment, referred to as the tumor microenvironment A variety of stromal cells, including T cells, B cells, natural killer (NK) cells, fibroblasts, adipocytes, vascular endothelial cells, and pericytes, surround the growing tumor. These cells secrete signals involved in tumor survival, growth, invasion, and migration, as well as change the behavior of cancer cells, also known as oncomodulation. TNF-α (tumor necrosis factor-alpha), mTOR (mammalian target of rapamycin), FASL (Fas ligand), PDL1/L2 (programmed cell death protein ligand 1/ligand2), TGF-β (transforming growth factor beta), STAT (signal transducer and activator of transcription), IL-1β (interleukin-1beta, EGF (epidermal growth factor), IL-6 (interleukin-6), sRAGE (soluble receptor for advanced glycation end product), IFN-γ (Interferon-gamma), HIF1A1α (hypoxia-inducible factor 1A1alpha), IGF2 (insulin-like growth factor-2), MMP14 (matrix metalloproteinase 14), VEGF (vascular endothelial growth factor), CXCL1 (C-X-C motif chemokine ligand 1), HMMR (hyaluronan mediated motility receptor), CXCR2 (C-X-C motif chemokine receptor 2), and PIGF (placental growth factor).

It has been reported that lung cancer development is related to chronic inflammation, described as infiltration of inflammatory cells and the accumulation of proinflammatory factors such as cytokines, prostaglandins, and chemokines that can stimulate various processes, including cell proliferation, angiogenesis, and metastasis [5]. Recent studies have reported that membrane receptors such as Toll-like receptors (TLRs), pattern recognition receptors (PRRs), and clusters of differentiation are capable of recognizing microorganisms, products of microbial activity, cytokines with proinflammatory roles, signaling molecules, proteins, and nucleic acids [6,7,8,9]. Furthermore, changes in the tumor microenvironment (TME) and the development of metastasis have also been observed [10]. In particular, the direct exposure to microbial oncogenes, toxins, and reactive oxygen species (ROS) from microbial activities can lead to mutations [7]. Moreover, the dysregulation of mechanisms of apoptosis, cell cycle regulation, and cell proliferation lead to carcinogenesis [11,12]. Therefore, developing a better understanding of the role of microorganisms in inflammation-induced cancer may prospectively lead to the development of antimicrobial therapies against cancer initiation and/or progression (Figure 1).

Ideally, an immune system is capable of identifying and destroying malignant cells. In order to evade host immune-mediated surveillance, tumors have developed several mechanisms that may involve the upregulation of inhibitory immune checkpoints, extension of local immunosuppressive microenvironments, and triggering of dysfunctional T-cell signaling [13]. In fact, macrophages produce proinflammatory cytokines, such as TGF-β, IL-6, IL-10, and TNF-α, that induce stem cell-like characteristics in tumor cells, thereby sustaining these tumor cells and allowing them to continue to grow [10].

## 2. Immune System Responses to Infectious Factors Leading to Tumorigenesis

**Figure 2:**
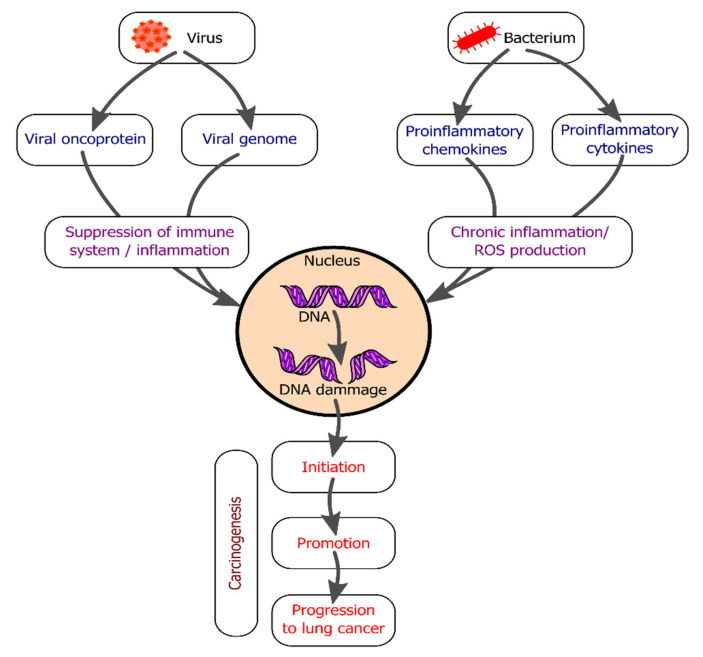
Overview of the role of bacteria and viruses in the development of lung cancer.

The immune system plays a pivotal role in the process of tumorigenesis via disturbance of the equilibrium of the immune system homeostasis by either promoting or inhibiting cancer cell proliferation [14,15]. Increasing numbers of studies have identified various infectious agents that are directly linked to the incidence of cancer and are deemed oncogenic [16,17]. Furthermore, it has been reported that inflammation induced by bacteria and viruses can increase the cancer risk [18] (Figure 2). Moreover, infectious organisms are capable of interfering with the transmission of oncogenic factors via either partial cross-immunity or immune assistance [15].

It is common knowledge that infections can occur during the first days of life, thereby testing the fragile immune system of the newborn. Interestingly, a newborn’s immunity is marked by a significant reduction of T-helper (Th)1 activity and an excess of Th2 activity. Therefore, increased exposure to bacterial and/or viral infections following birth is fundamental for the transformation of a Th2-biased immunity into a balanced Th1/Th2 immunity that is accompanied by immunological memory development. In particular, Th2 cells are capable of producing IL-4, which, in turn, can exploit an associated heteroreceptor to induce the apoptosis of Th1 cells [19]. Likewise, chronic inflammation associated with infections in children or adults is reported to precede tumor development [12]. Thereby, such an inflammation participates in carcinogenesis via the induction of oncogenic mutations and increased angiogenesis [15]. Moreover, such an inflammation will contribute to an enriched TME with innate immune cells, including natural killer cells, neutrophils, macrophages, dendritic cells, and myeloid-derived suppressor cells, as well as in adaptive immune cells such as B and T lymphocytes [20]. It is important to point out that particular cytokines, including IL-6, IL-17, and IL-23, are capable of either promoting or inhibiting cancer cell proliferation, as well as others, including IL-12, TRAIL, and IFN-γ, in contributing to the development and progression of a tumor [17]. Thus, the capacity of controlling the immune system to either promote or inhibit tumor progression is due to the activation of different downstream effectors such as caspases, SMAD 1 and SMAD 2, NF-κB, and AP-1 [21]. In fact, infections can play important roles at the early stages of tumorigenesis and during immunosurveillance by activating T and NK cells [22]. Interestingly, immunosurveillance and tumor-promoting inflammation may coexist even within the same tumor [23].

For the initiation of an antitumoral response, it is necessary that tumor-associated antigens (TAAs) are recognized by the immune system. Therefore, cell receptors can transport TAAs to T-helper (Th) lymphocytes from the lymph nodes that activate T cells and macrophages to kill cancer cells [24,25]. By increasing the exposure to antigens, a longer lifespan may induce chronic low-grade inflammation, thus contributing to immune disorders [26]. In turn, this may lead to an accumulation of cancer cells in older individuals [26]. Based on the current data from studies on respiratory tract infections, it is those infections occurring later in life that can play critical roles in the capacity of an immune system to control tumorigenesis [24]. For example, exposure to higher levels of environmental factors, such as endotoxins from the dust of dairy industry activities, can offer protection against lung cancer, whereas such protection is reduced following the removal of exposure [27,28].

In vivo studies have demonstrated that influenza viruses produce TAAs, inducing immune memory and a lifelong immunosurveillance of cancer cells [29]. Furthermore, common respiratory tract infections are also reported to be associated with an increased risk of chronic lymphocytic leukemia [30]. Interestingly, recent in vivo studies have found that type 2 inflammatory conventional dendritic cells (infcDC2s) are involved in respiratory viral infections [29]. Such DCs are observed to produce optimal prime CD4+ and CD8+ T-cell immunity in a type I (IFN)-dependent manner [31]. Immunologically, the analysis of tumor microenvironment inflammation has revealed an incidence of strong antigen and T-cell activation, leading to the development of tumor-specific CD8+ T cells capable of eliminating cancer cells and in developing long-term antitumor memory responses [32]. Moreover, proinflammatory γδ T cells have been identified to secrete a number of cytokines critical during microbial infection in the lungs. Thus, cytokines such as IFN-γ, TNF-α, IL-1β, and IL-17A enable the recruitment of innate mononuclear cells and stimulate microbial clearance [33,34,35].

### 2.1. Bacterial Infections and Lung Cancer Development

Many bacteria have been reported to have the capacity to alter various pathways and molecules of host cells in order to safeguard their intracellular survival. It is common knowledge that the lungs are associated with a sterile space. In recent studies, it has been found that alterations in the lung microbiome could contribute to disease states such as the aggravation of chronic obstructive pulmonary disease (COPD) [36,37]. In fact, it is reported that lung microbiota dysbiosis is correlated with the development of lung cancer [38]. For example, the oral taxa of *Streptococcus* and *Veillonella* are associated with the upregulation of the extracellular signal-regulated kinase (ERK) and the phosphoinositide 3-kinase (PI3K) inflammatory signaling pathways [39]. Therefore, it is important to go over some of the key pathogenic organisms that are either associated or involved in lung cancer development. The immune mechanisms of the different pathogenic bacterial organisms involved in lung cancer are presented in Table 1.

#### 2.1.1. *Chlamydophila pneumonia*

The bacterial pathogen *Chlamydophila pneumoniae* is reported to be associated with lung cancer, and different mechanisms have been proposed to explain such an association. It is proposed that one of the potential mechanisms of this association may be mediated via the generation of ROS during inflammation, thus leading to DNA damage [57]. It is reported that lung inflammation can increase the rate of cell division and the risk of incidence of a mutation through a fixed rate of DNA damage, thereby promoting cancer development [58]. In fact, effector molecules derived from *C. pneumoniae* are observed to elicit changes within the internal environment of host cells with the induction of immunosuppression, occurrence of chronic inflammation, inhibition of tumor suppressor mechanisms, and transformation of cells via oncogene transfer [39,40,57,58]. Moreover, during chronic lung infection, the proteins of *C. pneumoniae* produced in host cells are found to be capable of migrating to different organelles such as the nucleus, endoplasmic reticulum, and mitochondria, thereby influencing various important biological activities and ultimately leading to the development of cancer [40,58,59,60]. In addition, *C. pneumoniae* is found to be an effective inducer of IL-6, TNF-α, and IL-1β in host monocytic cells that may potentially contribute to carcinogenesis [40,41].

It has been reported that *C. pneumoniae* easily infects the lungs of smokers, thus increasing their risks for lung cancer [61]. It has long been established that smoking decreases the lung immunity and increases IL-4 secretion. Moreover, *C. pneumoniae* downregulates apoptosis by inducing IL-10, thereby leading to chronic infection [42,43]. Furthermore, chronic infection with *C. pneumoniae* may lead to the release of an endotoxin-like protein, chlamydial heat shock protein-60 (CHSP-60), that plays a key role in lung carcinoma pathogenesis [44]. Nevertheless, several proteins are reported to be released following *C. pneumoniae* infection, thus contributing to lung cancer as a result of competitive inhibition between the target proteins and host proteins for binding with their respective substrates [62]. In addition, *C. pneumoniae* infection results in nitric oxide production [63].

#### 2.1.2. *Mycobacterium tuberculosis*

The increased lung cancer incidence is highly related to the immunosuppression status as a result of *Mycobacterium tuberculosis* (MTB) infection [64]. Recent studies have identified inflammation and pulmonary fibrosis, both caused by tuberculosis, as major factors in lung cancer development [65]. It has been observed that inflammation associated with infections can promote carcinogenesis, leading to host tissue disturbance, the development of fibrosis or scar tissue, and genetic alterations [66,67,68]. *Mycobacterium tuberculosis* determines the activation of neutrophils, thereby producing ROS that can bind to DNA, which, in turn, can result in genetic damage and contributes to lung carcinogenesis [45,46].

Tuberculosis is associated with both lung squamous cell carcinoma and adenocarcinoma [69,70,71], leading to the release of inflammatory mediators such as IL-1, IL-2, and IL-12; tumor necrosis factor alpha (TNF-α); and INF-γ, which induce the inflammation of lung tissues [47,48]. On the one hand, extracellular matrix (ECM) components are produced so that they can participate in the tissue repair process, which is characterized by high activity levels of fibroblasts and increasing levels of TGF-β, IL-4, IL-10, IL-3, and IL-13 [47,48], whereas the inhibitory mechanisms such as immune evasion and immune checkpoint inhibition are involved in *M. tuberculosis* latent infections [51]. Therefore, type 1T helper cells (Th1) and the production of IFN-γ and TNF-α are activated in order to protect tissues/organs against *M. tuberculosis* infections. In a T-cell-mediated immune response, there is an interaction between the costimulatory and coinhibitory receptors from T-cell surfaces [72]. Recent data have demonstrated that a blockade of the programmed cell death protein 1 (PD-1)/programmed cell death ligand 1 (PD-L1) signaling pathway are able to prevent lung cancer development in patients with tuberculosis [72].

As to be expected, the timely and correct diagnosis of lung cancer is critical. However, the clinical symptoms such as dyspnea, chest pain, fever, hemoptysis, and weight loss, as well as radiological imaging, are similar for both lung cancer and tuberculosis disease [65,73]. It is observed that the induction of apoptosis and necrosis, as well as of tuberculosis reactivation, contributes to higher levels of IL-17 and THFα in immune-deficient patients. These higher levels of IL-17 and THFα may either reduce p53 (a tumor suppressor transcription factor) activity or increase Bcl-2 (B-cell lymphoma 2) expression, thus decreasing Bax-T (BCL2-associated X, an apoptosis regulator) and inhibiting caspase-3 due to lower levels of the expression of mitochondrial cytochrome C oxidase [49,50]. There is ample evidence that the Bacillus Calmette–Guérin (BCG) vaccine significantly increases the immune system response, as well as the levels of gamma interferon, nitric oxide, and interleukin-2 [64].

During the early stage of *M. tuberculosis* infection, intracellular mycobacterial death is avoided by the activation of an immune response with type 1T helper cells (Th1) and the secretion of both IFN-γ and TNF-α. Furthermore, it is reported that inhibitory mechanisms such as immune evasion and immune checkpoint inhibition support MTB latent infection [51]. In addition, the components of tuberculosis mycobacterial cells activate nitric oxide production and ROS, both playing roles in carcinogenesis [74]. Such a secretion of DNA-damaging reactive oxygen and nitrogen species by tuberculosis-infected macrophages, as a result of chronic tuberculosis infection, leads to the deletion of exon 19 of the epidermal growth factor receptor, an essential paracrine growth factor early in the process of carcinogenesis [75].

#### 2.1.3. *Cryptococcus* sp.

Pulmonary cryptococcosis is an invasive fungal infection, particularly incited by either *Cryptococcus neoformans* or *C. gattii* [76]. The involvement of *Cryptococcus* sp. in lung cancer development remains controversial, while Harada et al. proposed that coexisting cryptococcosis and lung malignancy are coincidental [77], Robinson et al. proposed that lung cancer development is the result of an immune suppression that predisposes a patient to *Cryptococcus* infection [78]. It is important to point out that the pulmonary is often misdiagnosed as a neoplasm, as radiologic images very well mimic a pulmonary neoplasia [76]. Ordinarily, a *Cryptococcus* infection results in a reaction by the host organism, manifested by the induction of Th1/Th17 immune responses, along with the activation of macrophages, changes in the proinflammatory cytokine expression, and activation of inflammatory dendritic cells [52]. An excessive inflammation and a powerful Th1/Th17 response can induce severe damages of the host organism [79,80].

#### 2.1.4. *Helicobacter pylori*

The Gram-negative spiral-shaped bacterium *Helicobacter pylori* is classified as a Group 1 carcinogen (as of 1994) by the International Agency for Research on Cancer (IARC). This is due to the fact that *H. pylori* is highly involved in the pathogenesis of functional dyspepsia, peptic ulceration, gastric adenocarcinoma, and mucosa-associated lymphoid tissue lymphoma (MALT) [55,81]. In extra-digestive pathologies, the immune and inflammatory responses are activated by *H. pylori* infection [82]. Furthermore, it has been suggested that *H. pylori* infection may also indirectly increase the risk of respiratory diseases by means of systemic inflammatory and/or autoimmune responses and/or by the aspiration of *H. pylori* products, such as exotoxins, into the lungs [53]. Various functional mechanisms such as cytotoxin-associated antigen A (CagA)-associated mechanisms and Src/p130cas signal cascades can explain *H. pylori*’s involvement in lung cancer [83]. Moreover, both the DNA and proteins from *H. pylori* have been identified in bronchoalveolar lavage from lung cancer and in lung biopsies [54,84,85]. For example, it has been observed that vacuolating cytotoxin (VacA) induces IL-6 and IL-8 production in lung carcinoma cells and IL-8 synthesis in bronchial epithelial cells, thus demonstrating a lung epithelium response to the pathogenic factors derived from *H. pylori* [53,54].

Clinical and experimental studies have indicated that *H. pylori* may reach the stomach due to aspiration into the tracheobronchial segment of some components of the stomach’s contents, such as bile acids and pepsin, thereby resulting in lung damage [84,85]. The lung inflammatory response is characterized by the overexpression of pathogen recognition receptors (PRRs) such as Toll-like receptors (TLRs) in order to identify pathogen-associated molecular patterns (PAMPS) [86]. Moreover, bacterial DNA can be identified by cytoplasmatic surveillance receptors such as TLR-9 [55] and by the receptors of advanced glycation end products (RAGE) [87]. Interestingly, recent studies have found that TLRs and RAGE can influence both the recruitment and activation of immune cell responses in lungs via the following two mechanisms: (1) directly by recognizing the PAMPs and (2) indirectly through the recognition of damage-associated molecular patterns (DAMPs) as a result of lung injuries [56].

### 2.2. Viral Infections and Lung Cancer Development

There are a number of viruses that are involved in lung cancer development. A description of these viruses, along with the mechanisms of the immune responses involved in lung cancer development, will be discussed (Table 2).

#### 2.2.1. Human Immunodeficiency Virus (HIV)

The human immunodeficiency virus has not been implicated in oncogenesis, but in HIV-positive patients, other associated infections may yield a chronic inflammatory state that can be involved in lung cancer carcinogenesis [83,88,95,96]. For example, for those HIV patients who smoke, there is a 2.5-fold increase in their risk of lung cancer [97]. Moreover, the increased risk of lung cancer in HIV patients is correlated with a low CD4 cell count, viral load, and increased bacterial pneumonia episodes [98].

Among various forms of cancer, lung cancer is the main cause of death among HIV-positive patients [99]. The development of lung cancer is linked with different factors, such as immunosuppression, CD4 count, and viral load, wherein immunosuppression is responsible for the observed higher rates of lung cancer in HIV patients [88]. As mentioned above, the risk of lung cancer increases with smoking tobacco, and among all adults, those with HIV are the least likely to quit smoking [100,101].

HIV infection negatively modulates the immune system, thereby leading to chronic inflammation and increasing risks of coinfections with other viruses, thus increasing the risk of cancer development [97,102]. In a large clinical study of HIV patients conducted in Montreal (Canada) from 1988 to May 2018, a high proportion of lung cancers are detected at very late stages of the disease. The metastatic disease is identified in 52% of patients [103].

#### 2.2.2. Human Papilloma Virus (HPV)

The human papilloma virus belongs to the Papillomaviridae family of DNA viruses. It has been observed that HPV has a high preference for invading epithelial tissues, such as squamous epithelium, bronchus, and lung. It is hypothesized that epithelial tissue damage may allow the virus to infect undifferentiated cells from the basal layers of a stratified squamous epithelium. The HPV life cycle and gene expression are controlled by epithelial cell differentiation [2]. Infection with HPV has been identified as responsible for approximately 5% of the global cancer burden [104,105]. Moreover, HPV infection has been reported as a risk factor for lung cancer development, particularly for patients infected with high-risk serotypes 16 and 18, nonsmokers, and females [106].

Although a number of studies have reported a link between HPV infection and lung cancer, this correlation remains controversial. HPV infection involvement in the pathogenesis of lung cancer in never smokers is deemed a major risk factor for such patients [83]. In ~20% of lung cancer cases, HPV DNA has been detected [106].

It is reported that, once the virus enters the lungs, it takes over the entire cellular mechanism, replicates its genome, avoids cell apoptosis, and initiates tumor formation [89]. A crosstalk between estrogen, hypoxia-inducible factor-1α (HIF-1α), and the epidermal growth factor receptor (EGFR) can activate mitogenic signaling, thus contributing to cell survival [89]. Recent studies have suggested that many signaling pathways related to lung cancer are altered by HPV. For example, the HPV E6 and E7 oncogene proteins are capable of regulating the gene expression of various target genes and proteins, such as p53, IL-6, IL-10, pRb, EGFR, HIF-1α, Mcl-1, Bcl-2VEGF, and cIAP-2, to promote lung cell proliferation, angiogenesis, and tumor progression [2]. Furthermore, the rate of HPV infection is found to be higher for squamous cell carcinoma than that for adenocarcinoma [107,108]. In addition, HPV infection induces inflammation and epithelial–mesenchymal transition (EMT), thus further suggesting its involvement in the development of lung cancer [90].

#### 2.2.3. Epstein–Barr Virus

The Epstein–Barr virus (EBV) has long been hypothesized to contribute to a number of lymphoproliferative and neoplastic disorders, such as gastric cancer, Hodgkin’s disease, and Burkitt’s lymphoma [109]. A strong association between EBV and lymphoepithelioma-like carcinoma, a rare form of lung cancer, has been detected in Asian patients but not in Western patients [110]. In a clinical study of 53 patients with lung cancer, EBV has been identified in the bronchoalveolar fluid, thus supporting the proposition that the lung tissue can serve as a potential EBV reservoir [111].

The relationship between EBV and lung cancer remains highly controversial, particularly due to the small sample size and limitations of traditional viral screening methods, such as PCR. In a study using microarray and real-time quantitative PCR (qPCR) analysis for three EBV miRNAs, it was found that both the microarray and qPCR detected either mature miRNA or pre-miRNA expression in some lung cancer cases; however, the pre-miRNA levels could not be correlated with mature miRNA levels for the lung cancer settings [112]. Interestingly, an in situ hybridization analysis detected EBV-encoded RNA (EBER) in non-small lung cancer cells [91]. In addition, an increased immune cell infiltration was detected by comparing samples with high levels of EBV transcripts to samples with low EBV transcripts. Furthermore, the activation of oncogenic pathways and prevalence of the inhibition of immune pathways for samples with high EBV transcripts were detected using next-generation sequencing (NGS), thus demonstrating the direct regulation of tumor pathways by EBV [91].

#### 2.2.4. Cytomegalovirus

Recent data have suggested that the cytomegalovirus (CMV) may have an oncomodulatory role, as it can stimulate cell cycle progress and increase cell proliferation for some cancer cells via the production of viral proteins that can influence DNA replication and gene expression [113]. It is observed that CMV may aid tumor cells in avoiding immune responses by preventing activated NK and T cells from killing cancer cells [92]. In fact, CMV infection has also been reported to increase tumor invasiveness by promoting the migration of infected cancer cells [92]. Furthermore, cyclo-oxygenase 2 (COX-2) is overexpressed in cancers of the colon, breast, prostate, and lung, and its inhibition blocks the replication of CMV [114,115].

In vivo studies have identified caspase activation in a p53-independent manner in the lung tissues of xenografted mice injected with HepG2 cells infected with wild-type (WT) CMV [116]. This indicates that apoptosis induction is not completely restricted to the tumor tissues of mice subcutaneously injected with CMV-infected HepG2 cells [116].

#### 2.2.5. Influenza Virus

In a study of cohorts, it is reported that exposure to influenza is correlated with a 1.09-fold increased risk of lung cancer and a 25% increased risk in patients with repeated episodes (5+) of influenza infection [117]. Moreover, the annual influenza vaccination administration in patients with chronic obstructive pulmonary disease may trigger a TH1 immune response, thus reducing the lung cancer risk [110]. Furthermore, recent studies have proposed that seasonal influenza vaccines are useful in the prevention of infection but, most importantly, in cancer immunotherapy. Thus, the injection of an influenza vaccine intratumorally promotes systemic CD8+ T-cell-mediated antitumor immunity and reduces tumor growth [32].

#### 2.2.6. Measles Virus (MV)

Measles is a ubiquitous RNA virus that may cause persistent viral infection, likely due to a mutated virus [118]. A ubiquitin E3 ligase, Pirh2, is overexpressed in lung cancer cells, and it is associated with p53 inactivation [112]. The expression of Pirh2 is an indicator of improved survival; however, MV phosphoprotein inhibits Pirh2 ubiquitination [119].

Interestingly, CD46 is a cell membrane complementary inhibitory protein that acts as an MV receptor, and it is overexpressed in lung cancer cells [93]. MV is demonstrated to be oncolytic for non-small cell lung carcinoma, and it is independently expressed in nectin-4. Furthermore, intratumoral injections of carcinoembryonic antigen (MV-CEA) promote tumor growth inhibition and detection of the viral transgene in mice sera [120].

#### 2.2.7. Severe Acute Respiratory Syndrome Coronavirus 2 (SARS-CoV-2)

Often, a lung cancer diagnosis is delayed due to similarities between the symptoms of lung cancer and SARS-CoV-2 infection, thus leading to further injuries of lung tissues [121]. An excessive inflammatory response to SARS-CoV-2 is correlated with high levels of circulating cytokines, acute lymphopenia, and significant mononuclear cell infiltration in the lungs [122]. In patients with severe COVID-19, similarities between the systemic cytokine profiles and cytokine release syndromes have been reported. In particular, it is observed that increases in the levels of IL-6, IL-7, and tumor necrosis factor (TNF) are accompanied by increases of inflammatory chemokines, such as CXC-chemokine ligand 10 (CXCL10), CC-chemokine ligand 2 (CCL2), and CCL3 [94]. Moreover, Type I interferons can induce the expression of SARS-CoV-2 entry receptors, thereby allowing the virus to migrate to the cytoplasm of a macrophage, promoting the activation of NLRP3 inflammasome and the secretion of mature IL-1β and/or IL-18. IL-1β is capable of promoting the activation of monocyte-derived macrophages and of reducing type I interferon production in the lungs [94]. Furthermore, the SARS-CoV-2 spike (S) protein binds to angiotensin-converting enzyme 2 (ACE-2), thus promoting penetration of the virus into cells along with host proteases, particularly of transmembrane serine protease 2 (TMPRSS2) [123].

## 3. Infectious Complications of Lung Cancer

Following acute lung damage, TNF-α, IL-1β, IL-8, and IL-6 are the first to appear, as they are highly active mediators of inflammatory pathways, while the anti-inflammatory cytokines are detected later [52]. Patients affected by non-small cell lung cancer (NSCLC) have a higher risk for recurrent infections that can be related to the oncological disease itself but, also, with cooccurring factors such as old age, smoking history, the presence of chronic obstructive pulmonary disease (COPD), and immunosuppression related to lung cancer therapies [3,124].

The lung mucosal tissue is colonized by a diverse bacterial community, and this is correlated with clinical outcomes in lung cancer patients, as this colonization causes lung adenocarcinoma-related inflammation via the stimulation of lung resident γδ T cells [125]. Often, *Pseudomonas aeruginosa*, a Gram-negative bacterium, is frequently found in the lungs of cancer patients, and this is accompanied by other factors, such as age-related comorbidities, aggressive tumors, and a rapid clinical decline [126]. In cancer patients, the risk for tuberculosis may be due to immunosuppression from the malignancy itself or from chemotherapy due to metastasis and, also, to structural alterations of the lung [127]. The incidence of lung cancer in a field of inactive pulmonary tuberculosis stimulates the reactivation of *M. tuberculosis* [128].

Immunotherapy with immune checkpoint inhibitors (ICIs) may disrupt T-cell-mediated immunity that produces excessive inflammation. Thus, the tissue from an MTB-infected environment is destroyed as a result of the reactivation of MTB [129,130]. In fact, MTB activation occurs in response to the activation of particular immune cell subsets in NSCLC patients receiving ICIs [131]. Moreover, interferon-γ release assays (IGRAs) are negative prior to treatment in patients with *M. tuberculosis* infection treated with nivolumab for stage IV lung cancer, but these assays turn positive following the eight cycles of nivolumab treatment [132].

Tuberculosis infection was reported in two patients treated with ICIs, wherein one of the patients also had metastatic melanoma [133]. These patients were treated with pembrolizumab and nivolumab for melanoma and lung cancer, respectively; however, ICI therapy was continued with the melanoma patient, but it was stopped with the lung cancer patient who died from spinal cord compression [134]. In another study, a patient with NSCLC who developed pulmonary tuberculosis during treatment with the anti-PD-1 agent nivolumab had a paradoxical response (PR) ten days after initiation of the anti-MTB treatment [132]. This demonstrated that anti-PD-1 agents enhanced the development of pulmonary TB, as well as the incidence of PR following anti-MTB treatment via upregulation of the immune response [135]. In another study using the Lewis lung carcinoma mouse model *Toxoplasma gondii*, infection inhibited tumor growth via the induction of both Th1 immune responses and antiangiogenic activity [136].

Of particular interest, cancer patients have been reported to be more susceptible to COVID-19 infection compared to individuals without cancer, and this is attributed to either a systemic reduced immunity or anticancer therapy [137]. Moreover, this finding is distinctly observed in lung cancer patients due to the fact that they already have chronic pulmonary inflammation [138]. Furthermore, lung cancer is the most common form of cancer in patients with COVID-19 infection [139,140,141]. This increased risk of infection in lung cancer patients may also be due to the abundance of viral spike protein-binding receptors that become available to host cells in the lungs, wherein angiotensin-converting enzyme-2 (ACE2) receptors are expressed on lung capillaries [142].

The immune system in lung cancer patients can lose the ability to control the proliferation or metastasis of tumor cells in HIV infections, as this is linked to a poor immune regulatory function [143,144].

## 4. Conclusions

A key element in a successful clinical practice is dependent on the early detection of various pathogens, particularly for lung cancer patients, as they are susceptible to attacks by various aggressive pathogenic bacteria and viruses. Moreover, these patients are highly dependent on a number of treatments, including immunotherapy; thus, this combination of treatments may lead to modulation of the systemic immune response. The infectious agents may not be the root causes of lung carcinogenesis, but they may facilitate the development of an inflammatory environment prone to lung cancer initiation and progression, as well as the response to therapy. In addition, there are reported associations between infectious factors and lung cancer. However, further studies are needed not only to confirm such associations but, also, to better understand the molecular mechanisms involved in these associations, as well as the contributions of other factors, such as smoking habits and air pollution, among others.

## Figures and Tables

**Table 1:** Immune mechanisms involved in lung cancer development by various bacterial pathogens.

Bacteria Inducing Lung Cancer	Effect(s) on the Immune System	Reference(s)
*Chlamydophila pneumoniae*	Induction of TNF-α, IL-1β, and IL-6	[40,41]
	IL-10 induction	[42,43]
	Release of CHSP-60 protein	[44]
*Mycobacterium tuberculosis*	Activation of neutrophils and production of reactive oxygen species	[45,46]
	Release of TNF-α, INF-γ, IL-1, IL-2, and IL-12	[47,48]
	Increase in levels of TGF-β, IL-4, IL-10, IL-3, and IL-13	[47,48]
	Increased levels of IL-17 and THFα	[49,50]
	Secretion of IFN-γ and TNF-α	[51]
*Cryptococcus* sp.	Activation of Th1/Th17 immune responses	[52]
*Helicobacter pylori*	Induced IL-6 and IL-8 production	[53,54]
	Overexpression of Toll-like receptors (TLRs)	[55,56]

**Table 2:** Immune mechanisms involved in lung cancer development by viruses.

Virus Inducing Lung Cancer	Effect(s) on the Immune System	Reference(s)
Human immunodeficiency virus	CD4 count	[88]
Human papilloma virus	Activation of the mitogenic signaling	[89]
	Increase TNF-α and reactive oxygen-nitrogen species (RONS)	[90]
	Activation of p53, IL-6, IL-10, pRb, EGFR, HIF-1α, Mcl-1, Bcl-2VEGF, and cIAP-2	[2]
Epstein–Barr virus	Increase immune cell infiltration	[91]
Cytomegalovirus	Prevention of activated NK and T cells	[92]
Influenza virus	Promotion of systemic CD8+ T cell-mediated antitumor immunity	[32]
Measles virus	Overexpression of CD46	[93]
SARS-CoV-2 virus	Increase IL-6, IL-7, TNF-α, CCL2, CCL3, and CXCL10	[94]
	Secretion of mature IL-1β and/or IL-18	[94]
